# The prognostic value of the Barthel Index for mortality in patients with COVID-19: A cross-sectional study

**DOI:** 10.3389/fpubh.2022.978237

**Published:** 2023-01-24

**Authors:** Erchuan Wang, Ao Liu, Zixuan Wang, Xiaoli Shang, Lingling Zhang, Yan Jin, Yanling Ma, Lei Zhang, Tao Bai, Jun Song, Xiaohua Hou

**Affiliations:** ^1^Division of Gastroenterology, Union Hospital, Tongji Medical College, Huazhong University of Science and Technology, Wuhan, Hubei, China; ^2^Division of Nursing, Union Hospital, Tongji Medical College, Huazhong University of Science and Technology, Wuhan, Hubei, China; ^3^Division of Respiratory and Critical Care Medicine, Union Hospital, Tongji Medical College, Huazhong University of Science and Technology, Wuhan, Hubei, China

**Keywords:** COVID-19, Barthel Index, prognosis, activities of daily living, decision tree

## Abstract

**Objective:**

This study aimed to analyze the association between the activity of daily living (ADL), coronavirus disease (COVID-19), and the value of the Barthel Index in predicting the prognosis of patients.

**Methods:**

This study included 398 patients with COVID-19, whose ADL at admission to hospital were assessed with the Barthel Index. The relationship between the index and the mortality risk of the patients was analyzed. Several regression models and a decision tree were established to evaluate the prognostic value of the index in COVID-19 patients.

**Results:**

The Barthel Index scores of deceased patients were significantly lower than that of discharged patients (median: 65 vs. 90, *P* < 0.001), and its decrease indicated an increased risk of mortality in patients (*P* < 0.001). After adjusting models for age, gender, temperature, pulse, respiratory rate, mean arterial pressure, oxygen saturation, etc., the Barthel Index could still independently predict prognosis (OR = 0.809; 95% CI: 0.750–0.872). The decision tree showed that patients with a Barthel Index of below 70 had a higher mortality rate (33.3–40.0%), while those above 90 were usually discharged (mortality: 2.7–7.2%).

**Conclusion:**

The Barthel Index is of prognostic value for mortality in COVID-19 patients. According to their Barthel Index, COVID-19 patients can be divided into emergency, observation, and normal groups (0–70; 70–90; 90–100), with different treatment strategies.

## 1. Introduction

Coronavirus disease (COVID-19) has grown rapidly and become a serious medical crisis that the whole world has had to confront (1–3). With the global trend of the epidemic, more than 500 million people are estimated to have suffered from COVID-19, with over 6 million people dead (4). The disease influences multiple organs and the overall function of the body, causing various symptoms including cough, breathing difficulties, headache, diarrhea, and muscle or joint pain (5). Some patients have severe symptoms such as stroke, seizures, and kidney failure (6–8), which greatly affect their activity of daily living (ADL). Meanwhile, the huge number of patients puts much pressure on medical staff and public health services (9, 10), and many patients have to take care of themselves. Therefore, the evaluation of ADL is a necessary part of clinical management. A quick assessment model of patients' prognosis is also required to stratify the patients and identify those at high risk of death, in order to promote the allocation of medical resources and reduce mortality and the burden on the medical system and society.

Some factors have been reported to be able to indicate the prognosis of patients, such as various routine laboratory parameters, cytokines, and features of lung computed tomography (CT) scans (11–16); great prediction models based on these factors have been developed, like the ISARIC score (17). However, these models are complicated, depend on additional clinical tests, and require further calculations. Given the large number of patients, a simple evaluation method that does not require any complex or expensive clinical trials would be valuable. ADL, which can effectively reflect patients' functional state by assessing their self-care ability, is simple to measure and practical. Notably, impaired ADL is shown to be a risk factor for death in COVID-19 patients (18), but its association with COVID-19 patients' prognosis has not been well-elucidated. Thus, a quick and concise prediction method based on it is needed.

The Barthel Index has been proposed as a prevalent tool for evaluating patients' ADL due to its reliability, sensitivity, and utility (19–21). It assesses patients' state in ten representative aspects of self-care ability, and has been widely utilized for clinical practices like predicting physical function alterations of geriatric rehabilitation patients (22), survival time of older patients (23), and functional recovery of stroke patients (24, 25). Whether it can be used as a prognostic predictor for COVID-19 has been rarely discussed. This study aims to evaluate the connection between ADL and COVID-19, and explore the value of the Barthel Index in predicting the prognosis of patients with COVID-19, further providing evidence for clinical treatment and management.

## 2. Materials and methods

### 2.1. Design

This retrospective cross-sectional study of COVID-19 collected simple health care clinical records of patients and intended to develop a simple evaluation method to predict the prognosis of the patients without the aid of any complex or expensive clinical tests. It adopted a convenient sampling method and included patients with COVID-19 who were hospitalized in a tertiary hospital in Wuhan from January 2020 to February 2020. Patients were divided into discharged or deceased groups according to their outcomes.

### 2.2. Participants

A total of 413 patients were retrospectively surveyed, while 398 were included in the final study. The inclusion criteria included: (1) patients diagnosed with COVID-19 by symptoms, polymerase chain reaction test, and other clinical examinations; (2) patients who completed the Barthel Index test. The exclusion criteria included: (1) patients who were younger than 18 years (*n* = 12); (2) patients who had serious organic diseases or lost self-care ability before onset (*n* = 3). The ethical approval of this study was waived by the local Ethics Committee of the Union Hospital, Tongji Medical College, Huazhong University of Science and Technology, in view of the retrospective nature of the study and that all the procedures being performed did not interfere with routine care.

### 2.3. Data collection

The sample size was calculated as follows. For regression analysis, the sample size should be 5–10 times the number of variables. All variables should be obtained directly and simply from the health care clinical records at admission without the use of other tools. Finally, 12 variables were collected. Therefore, at least 60 samples were required for both the discharged and the deceased groups. Meanwhile, the overall mortality in the early stages of the pandemic was 16.4% (26) to 20.3% (27). We took 18% as the estimated mortality, thus the total samples should be at least 333. In our study, 413 patients met the inclusion criteria and 15 patients were excluded. A final total of 398 patients were analyzed.

Individual assessments of the index were conducted by a trained team, strictly according to the standard of the index. The ADL of the patients in the present study were assessed on the day of admission to the hospital by Barthel Index, which includes feeding, bathing, grooming, dressing, bowel control, bladder control, toileting, chair transfer, ambulation, and stair climbing (28). Each item was divided into different grades of 15 (10), 5, and 0, depending on how much help they needed to complete the relevant items. Zero points meant patients could not do the relevant item independently and were completely reliant on help from others, while 15 (10) indicated that they did not need any help from others. Patients who needed a little or more help to complete the activities would be given scores somewhere between 0 and 15 (10). Then, the score of each item was added to get the final score, which had a range of 0–100. One hundred meant patients could take care of themselves without any help, while 0 meant they could not live without help from others. Finally, patients were given different scores to show their ability to perform daily activities.

Other information, including gender, age, temperature, pulse, respiratory rate, mean arterial pressure, oxygen saturation (SPO_2_), whether or not they could walk to the hospital, and total hospital days, were collected from patients to explore their association with the patients' prognosis as well. Additionally, the Braden score and Caprini score, which are usually used to assess the risk of pressure ulcers and venous thrombus embolism, were also recorded. All these variables reflected the patients' physical condition before receiving any treatment.

### 2.4. Data analysis

Firstly, in the analysis of the demographic characteristics, normality was tested using the Shapiro-Wilk test, and then, based on the result, variables were presented as median (25–75% interquartile range) or frequency (proportion). Differences of the continued variables between the discharged and the deceased were analyzed by *t*-test or Mann–Whitney *U* test, while differences of categorical variables were analyzed by chi-square test. Then, univariate logistic regression analysis was applied to test the correlation between the clinical characteristics and patient outcomes. As the minimum unit of measurement of the Barthel Index is 5, logistic regression analysis also explored the association between the index (5 points) and prognosis. The dose-effect relationship between Barthel Index and death risk was analyzed by the linear trend of the chi-square test. Moreover, the 100-Barthel Index was utilized to draw the receiver operating characteristic (ROC) curve to effectively show the relationship between the index and the prognosis of the patients. The area under the curve (AUC) was also measured and compared with the baseline area (0.5) to verify whether it was significantly different.

Additionally, in order to test the independent predictive ability of the Barthel Index, four regression models were constructed: (1) model 1 was adjusted for age and gender; (2) model 2 additionally included Braden score and Caprini score; (3) model 3 further included respiratory rate, SPO_2_, and whether or not patients could walk to the hospital; and (4) for model 4, the analysis was further adjusted for temperature, pulse, and mean arterial pressure. The added covariates were selected from the confounding factors, which were clinically associated with the prognosis of patients or statistically associated in univariate analysis (*p* < 0.05). Finally, a decision tree model was constructed by the method of split sample verification. All variables could be obtained from the health care clinical records, so there were no missing data to be addressed. SPSS version 25.0 (IBM) was used to analyze the data and Adobe Illustrator CC 2018 (Adobe Inc., Mountain View, CA, USA) was used to draw the figures. All *P*-values were two-tailed and *P* < 0.05 was considered statistically significant.

## 3. Results

### 3.1. Demographic characteristics

**Figure 1:**
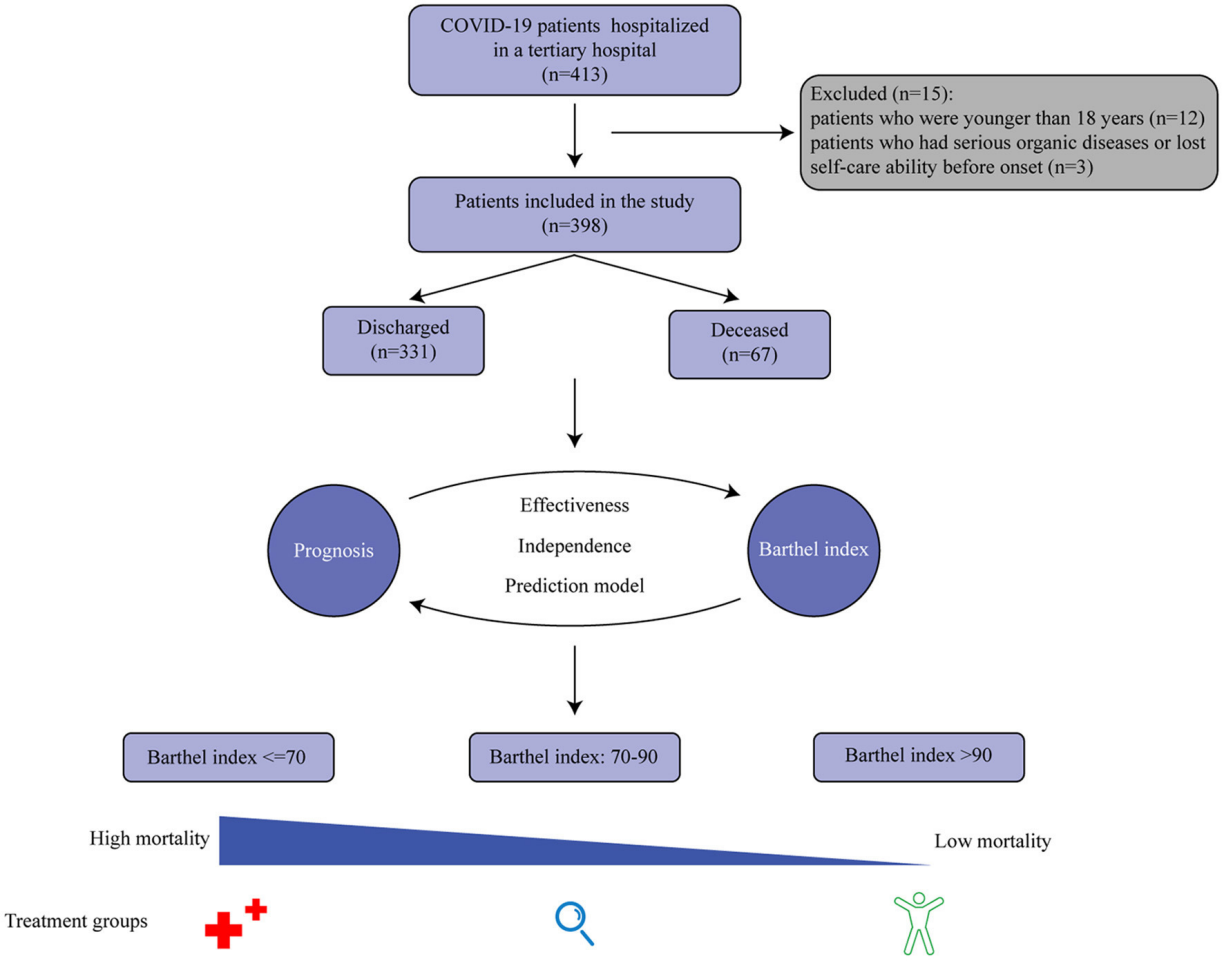
A graphical abstract of the study. The Barthel Index is significantly associated with the prognosis of COVID-19 patients, and a three-group classification scheme based on the index has been developed to facilitate disease management.

**Figure 2:**
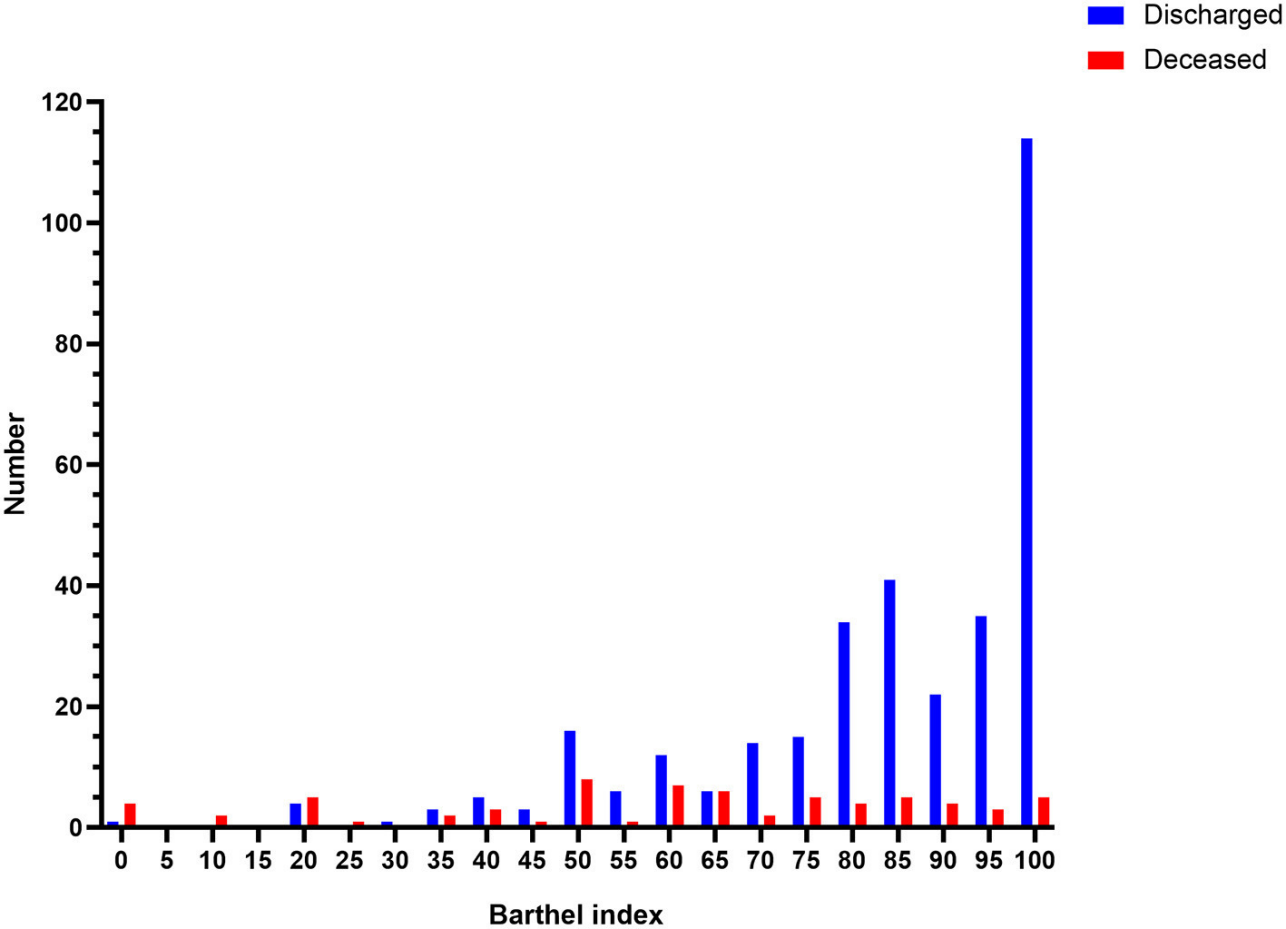
The distribution of the Barthel Index in the deceased and the discharged patients. The histogram shows the obvious difference in the distribution of the Barthel Index between the deceased and discharged patients. Deceased patients are presented on the (**Right**), while discharged patients are presented on the (**Left**).

In total, 413 patients were retrospectively reviewed. After excluding patients who were younger than 18 years (*n* = 12) and those who had serious organic diseases before onset (*n* = 3), the final study included 398 patients, consisting of 331 discharged patients and 211 male patients (Figure 1). The participants ranged from 20 to 96 years old, and the mean age was 58 years old (Supplementary Figure 1). Overall, 25.6% of patients could not walk to the hospital and half of the patients had an oxygen saturation higher than 95%. More information about the patients' characteristics are shown in Table 1. Notably, the median score of the Barthel Index in the discharged group (90) was higher than that in the deceased group (65), and the distributions of the Barthel Index in the two groups were dissimilar (Figure 2). In fact, the Barthel Index was significantly different (*P* < 0.001) between the discharged (mean rank = 218.64) and the deceased (mean rank = 111.93) groups.

**Table 1:** Demographic characteristics of the COVID-19 patients.

**Items**	**Discharged (*n* = 331)**	**Deceased (*n* = 67)**	***P*-value**
Gender^a^			0.001
Female	168 (50.8)	19 (28.4)	
Male	163 (49.2)	48 (71.6)	
Whether or not capable of walking to the hospital^a^			< 0.001
No	69 (20.8)	33 (49.3)	
Yes	262 (79.2)	34 (50.7)	
Age (years)	58 (46, 68)	67 (60, 76)	< 0.001
SPO_2_ at admission (%)	95 (93, 97)	88 (76, 93)	< 0.001
Temperature at admission (°C)	36.7 (36.4, 37.2)	36.7 (36.3, 37.8)	0.164
Braden score	21 (19, 23)	19 (16, 21)	< 0.001
Mean arterial pressure (mmHg)	96 (90, 103)	97 (87, 109)	0.678
Respiration	20 (20, 22)	22 (20, 26)	0.024
Pulse (/min)	88 (79, 99)	85 (77, 100)	0.811
Barthel Index	90 (75, 100)	65 (45, 85)	< 0.001
Caprini score	1 (0, 2)	2 (1, 3)	< 0.001
Total hospital days (day)	25 (17, 39)	8 (4, 15)	< 0.001

^a^These variables are presented as frequency (proportion), others are presented as median (quartile).

### 3.2. Barthel Index was a significant and independent indicator for the prognosis of the patients

We conducted logistic regression analysis to figure out the association between the prognosis and the diverse factors. Eligible factors (*P* < 0.05) included gender, age, temperature, respiration, oxygen saturation, whether or not patients could walk to the hospital, Barthel Index, Braden score, and Caprini score (Table 2). Among these factors, the Barthel Index was a potential indicator for its efficiency (per 5-point, OR = 0.817, 95%CI = 0.771–0.866, *P* < 0.001). In addition, the dose-effect relationship between the Barthel Index and the mortality risk of COVID-19 patients was analyzed, showing that a decrease in the Barthel Index indicated an increased risk of mortality (*P* < 0.001, by linear trend of the chi-square test).

**Table 2:** Univariate logistic regression analysis of the association between the factors and the prognosis of the patients.

**Factors**	**OR**	**95% CI**	***P*-value**
Gender			
Female			Reference
Male	2.604	1.468–4.619	0.001
Age	1.055	1.033–1.078	< 0.001
Temperature	1.531	1.128–2.078	0.006
Pulse	1.005	0.989–1.022	0.515
Respiration	1.117	1.049–1.190	0.001
Mean arterial pressure	1.004	0.984–1.024	0.703
SPO_2_	0.857	0.819–0.898	< 0.001
Whether or not capable of			
walking to the hospital			
No			Reference
Yes	0.271	0.157–0.469	< 0.001
Barthel Index	0.817	0.771–0.866	< 0.001
Braden score	0.793	0.725–0.867	< 0.001
Caprini score	2.703	1.835–3.981	< 0.001

To verify whether the Barthel Index could independently predict prognosis, four regression models were constructed by multivariable logistic regression. Model 1 showed the Barthel Index had a significant association with the outcome (Table 3, OR = 0.816, 95%CI: 0.763–0.873). Adjusting for Braden score and Caprini score (model 2), it remained a significant indicator for COVID-19 (OR = 0.814, 95%CI: 0.762–0.868). Model 3 revealed that including more factors only slightly weakened the association between the Barthel Index and the prognosis (OR = 0.825, 95%CI: 0.768–0.885). The consequences of model 4 (OR = 0.809, 95%CI: 0.750–0.872) showed that the Barthel Index was still a positive factor for survival and could be an independent indicator. Additionally, other independent predictors were also found in model 4, which included gender, temperature, pulse, and SPO_2_.

**Table 3:** Logistic regression models for Barthel Index as a predictor for the prognosis of COVID-19 patients.

**Logistic regression model**	**OR**	**95% CI**	***P*-value**
Model 1	0.816	0.763–0.873	< 0.001
Model 2	0.814	0.762–0.868	< 0.001
Model 3	0.825	0.768–0.885	< 0.001
Model 4	0.809	0.750–0.872	< 0.001
Other independent predictors in			
model 4			
Gender			
Female			
Male	3.813	1.767–8.228	0.001
Temperature	2.073	1.368–3.141	0.001
Pulse	0.974	0.950–0.997	0.030
SPO_2_	0.868	0.827–0.911	< 0.001

### 3.3. The prognostic prediction model of the Barthel Index for the patients

**Figure 3:**
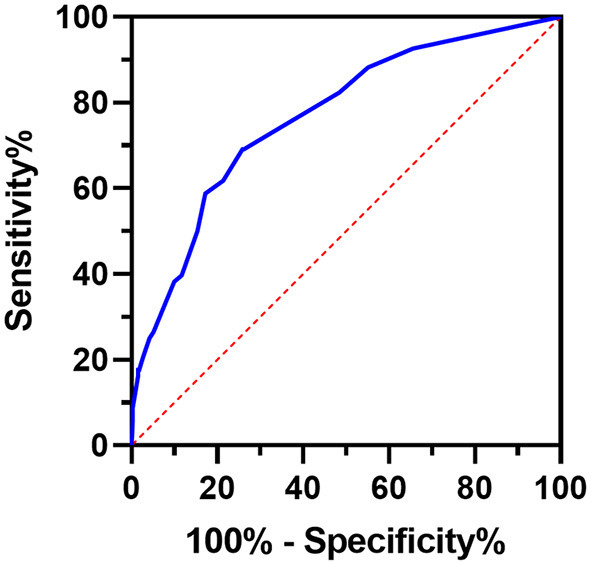
ROC curve of the Barthel Index in patients with COVID-19. ROC curve indicates the sensitivity and specificity of the Barthel Index in predicting the prognosis of patients with COVID-19. The AUC was 0.764 (95%CI: 0.701–0.827).

**Figure 4:**
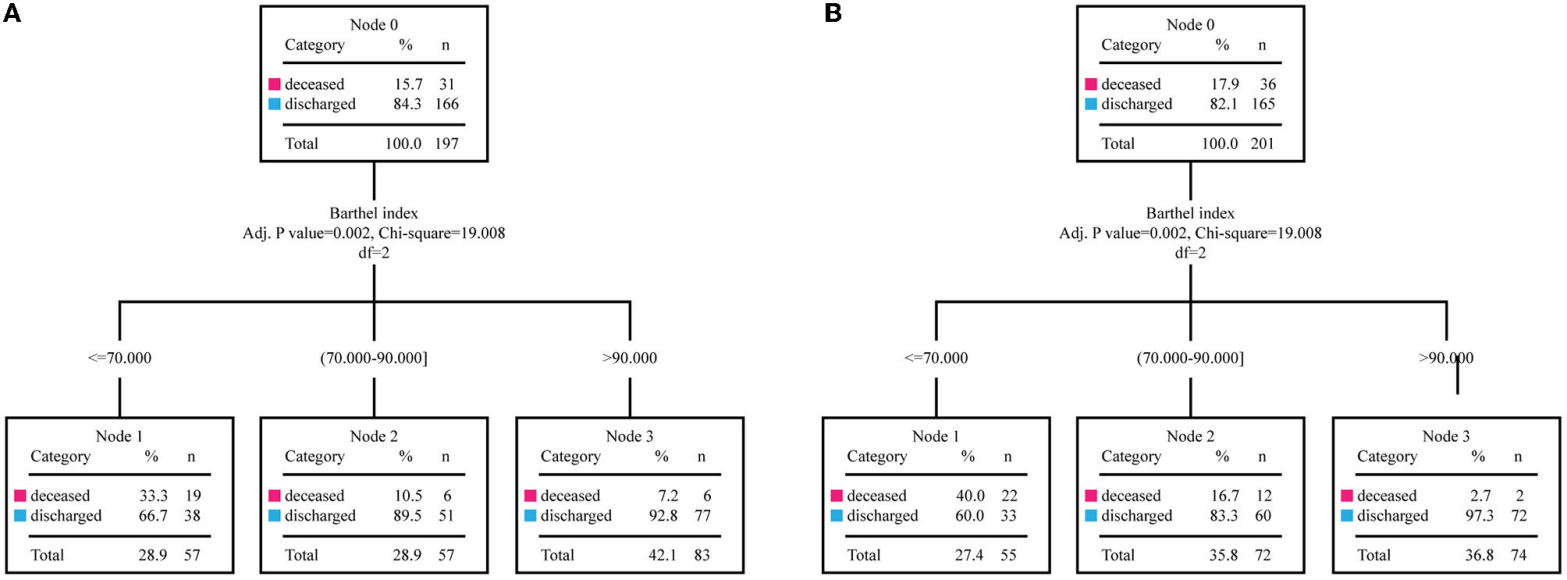
The training samples and the test samples of the decision tree. By means of the split sample verification, the entire study population is divided into the training samples (*n* = 197) and the test samples (*n* = 201). **(A)** The training samples of the decision tree are divided into three groups which had different mortality. **(B)** The divided groups in test samples show similar mortality with the corresponding groups in the training samples.

The Barthel Index and the outcomes of the patients were then used to draw the ROC curve (Figure 3). As a result, the AUC of the Barthel Index was 0.764 (95%CI: 0.701–0.827). When the Youden Index was maximum, the value of the Barthel Index was 76.5 and the sensitivity and the specificity were 0.740 and 0.687 (*P* < 0.001), respectively. To further improve the prediction efficiency, a prognostic prediction model was constructed by decision tree. By means of the split sample verification, all the subjects were randomly divided into training samples (Figure 4A) and test samples (Figure 4B). The training samples showed that patients whose Barthel Index were below 70 had a high risk of death, while people whose scores were above 90 were more likely discharged (mortality rates: 33.3 vs. 7.2%). The conclusion was still effective in the test samples (mortality rates: 40.0 vs. 2.7%).

## 4. Discussion

This cross-sectional study aimed to develop a rapid evaluation method that did not require any complex or expensive clinical trials to predict the prognosis of COVID-19 patients. In this study, we observed that the Barthel Index was significantly associated with the prognosis of COVID-19 patients and we developed a potential prediction model based on the index to facilitate disease management.

As a serious epidemic, COVID-19 impairs body function, can cause serious sequelae (28), and affects patients' ADL. ADL is closely associated with a patient's quality of life and has been used to evaluate the development of diseases like stroke (29). There are several tools to measure patients' ADL (30) and the Barthel Index, which estimates 10 items concerning functional state, is a simple and widely utilized one in, for instance, patients with neuromuscular or musculoskeletal disorders (28, 31). Notably, it has been reported that COVID-19 worsens all aspects of the Barthel Index in patients (32), and in this study the Barthel Index of deceased patients had also declined to a much lower level than the patients who were discharged. What is more, although the discharged patients also suffered from COVID-19, the Barthel Index in many of them remained at 100, which strongly indicates the index is a positive factor for the patients' survival. Thus, the Barthel Index can be of great value in disease management, and more attention should be paid to it.

Meanwhile, some other demographic characteristics of the discharged and deceased patients were also different. For instance, 79.2% of the discharged patients could walk to hospital, while only 50.7% of the deceased could do so. Notably, the univariate logistic regression analysis showed younger, female patients and those with high levels of SPO_2_ and low respiration rate had lower mortality. At the same time, the risks of pressure ulcers and venous thrombus embolism were lower in discharged patients than in the deceased patients, as measured by the Braden score and the Caprini score. Regarding the length of hospital admission, the discharged patients stayed in hospital much longer than the deceased (25 vs. 8 days), suggesting patients needed more time to recover and further illustrating that functional state or ADL, reflected by Barthel Index, is vital for patients. However, compared to the Barthel Index, these factors above could only partially reflect the patients' condition and were not suitable as evaluation tools for prognosis. In contrast, the Barthel Index could provide a comprehensive and effective evaluation of patients.

Furthermore, the Barthel Index showed great predictive value of prognosis by univariate logistic regression analysis, and had a negative relationship with mortality by linear trend of the chi-square test. To test whether it could be an independent indicator, different logistic regression models based on the Barthel Index were constructed, where many other factors that were clinically or statistically associated with the prognosis of COVID-19 were adjusted. Therefore, after constructing four models and finally adjusting for age, gender, Braden score, Caprini score, respiratory rate, SPO_2_, whether or not patients could walk to hospital, temperature, pulse, and mean arterial pressure, the Barthel Index still showed great value and could be independently utilized in the prediction of the prognosis.

Some other studies also found the Barthel Index was associated with the mortality of COVID-19 patients (33–35), but none of them offered a simple and practical evaluation method. Another study reported that the Barthel Index was not a significant predictor (*P* = 0.128), but that may be due to its small sample size (*n* = 146) (36). According to the ROC curve, the AUC of the Barthel Index was 0.764 (95%CI: 0.701–0.827), which was different from the reference (0.5), and the value of the Barthel Index was 76.5 when the Youden Index was maximum. Although the cut-off value could be used to predict prognosis, its sensitivity and specificity was not satisfactory. To further improve the prediction efficiency, a decision tree was constructed, by which we could conveniently use the Barthel Index to make a quick judgement concerning the risk of death of the patients. The decision tree established distinct classification schemes based on the Barthel Index values, and automatically compared their results to obtain the best scheme that could effectively distinguish patients with different outcomes. Meanwhile, the patients were randomly divided into training and test groups, and the classification scheme obtained from the training group was applied to the test group to verify its validity. Ultimately, in the training samples (*n* = 197), the high scorers (Barthel Index >90) had low mortality (7.2%) while the mortality of low scorers (Barthel Index ≤ 70) was quite high (33.3%). Moreover, the mortality of the remaining individuals (Barthel Index: 70–90) was between the two values (10.5%). Therefore, patients could be divided into three subgroups by the threshold 70 and 90: an emergency group (0–70), observation group (70–90), and normal group (90–100). Patients in the emergency group require all-round care as soon as possible, while patients in the normal group can receive routine treatment and are likely to be discharged after treatment. The observation group should be observed for longer. Applying the findings to the test samples (*n* = 201), we found that the model performed even better (mortality of emergency, observation and normal groups: 40.0, 16.7, and 2.7%). All in all, medical staff can quickly evaluate a COVID-19 patient's functional state with this model, and predict prognosis in order to provide them with appropriate and effective treatment.

There were other potential predictors of the patients' prognosis, including gender, age, temperature, respiratory rate, SPO_2_, whether or not they could walk to the hospital, Braden score, and Caprini score. However, after being adjusted in logistic regression models, only gender, temperature, pulse, and SPO_2_ had independent correlations with the disease. Braden score and Caprini score were also commonly featured in health care clinical records like the Barthel Index. In univariate logistic regression analysis, both were associated with the prognosis, while in the multivariate models, their association with the prognosis was not significant, indicating that other factors in the models were more relevant to prognosis. For other significant factors in model 4, they were either unstable or only partially reflected the patient's physical condition. In addition, the most frequently reported predictors of prognosis in COVID-19 patients were age, symptoms, comorbidities, and features derived from CT (15, 16, 37–40). A high proportion of severe cases and high mortality were observed in elderly COVID-19 patients (41, 42). But in our study, after being incorporated into the model with the Barthel Index, this no longer mattered (*P* = 0.139 vs. Barthel index: *P* < 0.001). Patients showed various symptoms, and some symptoms would also change over time, indicating that they were not appropriate or reliable indicators for all patients. Comorbidities or the relevant evaluation indicator, Charlson's Index, was associated with mortality (40). However, Charlson's Index was always concerned with long-term prognosis and could not provide a real-time view of a patient's functional state after being affected by COVID-19 like the Barthel Index. More importantly, the Barthel Index could more concretely reflect the effect of comorbidities on functional states and was also more accessible. Compared with the CT, the Barthel Index could be implemented on the spot and provided a quick assessment of patients, with features of comprehensiveness, test-retest, and easy execution. Some studies also showed that hypertension was a risk factor (43, 44), while our study and others indicated that after adjustment for other clinical and demographic parameters, hypertension was not an independent predictor of the prognosis (45, 46). To sum up, the Barthel Index has great predictive and practical value in the prognosis of COVID-19 patients.

This study has several advantages. First, prediction models based on clinical trials, like ISARIC score, have been well-reported, while there are few models based on care records. This study is a pioneer in demonstrating a rapid prediction model of the Barthel Index in the care records of COVID-19 patients. It uses only the information available at admission, rather than the results of other sophisticated clinical tests or tools. Moreover, the Barthel Index is easily accessible to health care workers, and its predictive value becomes even more precious when medical resources are scarce. Second, possessing a prediction for each patient in advance promotes the implementation of precision and personalized medicine to improve the therapeutic effect and the patient's feelings during treatment. Third, medical staff can distinguish critical cases from ordinary patients and treat them differently to reduce the mortality of the disease. Meanwhile, this model could reduce overtreatment of patients and also ease the burden on public health services and the economy. Therefore, this model can effectively help address this public health crisis, which affects millions of people and consumes massive public resources. Finally, the threshold of the index was determined in the training samples by the decision tree and verified in the test samples. Therefore, the conclusion was not affected by subjective factors and at the same time its practicability was proven.

On the other hand, there are still some limitations in the current study. Comparatively speaking, the index was not particularly effective in the observation group (Barthel index: 70–90), which indicates the outcomes of patients with a slight decrease in the Barthel Index are uncertain and different. Studies with larger sample sizes may help solve this problem. Secondly, the Barthel Index may begin to decline from the onset of the disease, and some studies have reported residual effects and persistent symptoms of COVID-19, such as fatigue, dyspnea, chest pain, cognitive disturbances, and a decline in quality of life after the discharge (47–49). Thus, the Barthel index should be evaluated before onset, at onset, and after rehabilitation to achieve dynamic results. By analyzing the alteration of the index, we can have a more comprehensive understanding of the disease. Thirdly, as a retrospective study, this study investigated the patients in the early stages of the pandemic, when the lethality of the disease was at its highest. The patients in our study were relatively affected and required hospitalization. Our findings were significant for them, but more studies are needed to understand the role of the Barthel Index in asymptomatic or mild cases. Additionally, there was selection bias in the process because we collected data from a single hospital and did not adopt the method of random sampling. Research involving multiple regions should be conducted in the future to verify the conclusion.

## 5. Conclusions

Assessed by the Barthel Index, the ADL of COVID-19 patients was significantly different between the discharged group and the deceased group. It was associated with the mortality of the patients, and the Barthel Index was a rapid and useful tool for predicting the prognosis of patients. The decision tree showed that patients with a Barthel Index below 70 had higher mortality, while those above 90 were commonly discharged after treatment. This is of value for guiding clinical management.

## Data availability statement

The datasets analyzed and generated during the current study are not publicly available due to the confidential policy of National Health Commission of China, but are available from Jun Song upon reasonable request.

## Ethics statement

The ethical approval of this study was waived by Ethics Committee of the Union Hospital, Tongji Medical College, Huazhong University of Science and Technology in view of the retrospective nature of the study.

## Author contributions

JS, TB, and XH designed the study. AL, TB, and EW performed the data analysis. AL and TB wrote the manuscript. JS and EW revised the manuscript. ZW, XS, LZ, YJ, YM, and LZ collected the data and conducted preliminary data processing. All authors contributed to the article and approved the submitted version.

## Supplementary material

Supplementary Figure 1 Age distribution of the participants in this study.
