# Supplementary figures and images for: The prognostic value of the Barthel Index for mortality in patients with COVID-19: A cross-sectional study
Source: Front Public Health. 2023 Jan 24;10:978237. doi: 10.3389/fpubh.2022.978237 (PMC9902915; doi:10.3389/fpubh.2022.978237)

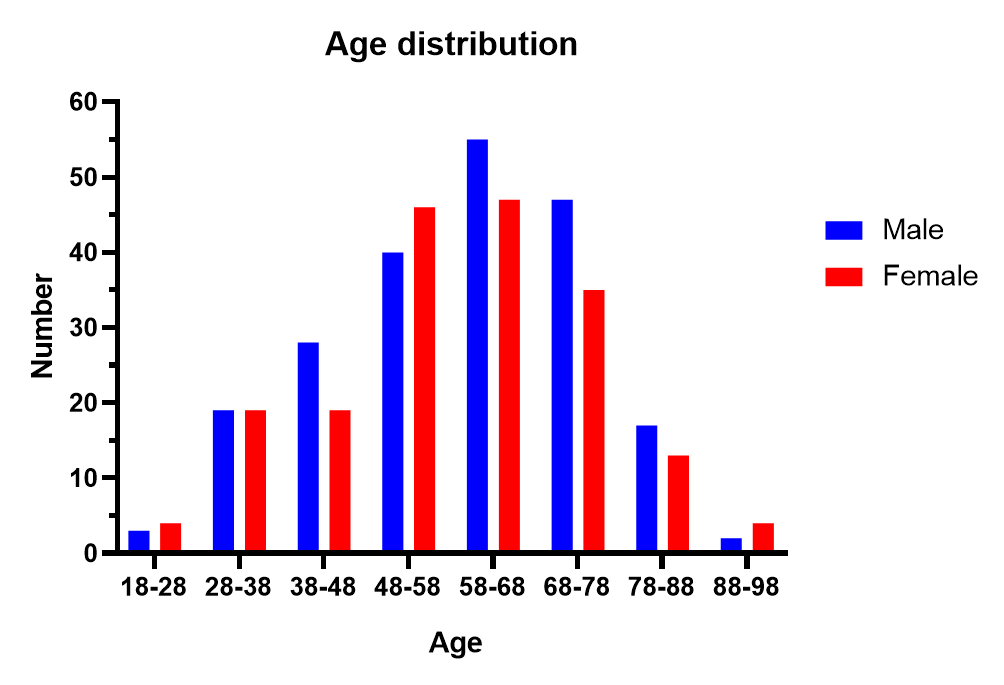

Supplement: Supplementary Figure 1 — Age distribution of the participants in this study. [file Image_1.TIF]
